# Older Adults’ Attitudes Toward Virtual Volunteering During the COVID-19 Pandemic

**DOI:** 10.1093/geroni/igaa057.3489

**Published:** 2020-12-16

**Authors:** Peter Sun, Nancy Morrow-Howell, Elizabeth Pawloski, Emma Swinford

**Affiliations:** 1 Washington University in St. Louis, Saint Louis, Missouri, United States; 2 Washington University in St. Louis, St. Louis, Missouri, United States; 3 Oasis Institute, St. Louis, Missouri, United States; 4 University of Missouri-Kansas City, Kansas City, Missouri, United States

## Abstract

This study explored older adults’ attitudes toward virtual volunteering during the COVID-19 pandemic. A 22-item survey was administered to 229 volunteers who previously worked with children through the Oasis Intergenerational Tutoring program in St. Louis. Questions focused on technology use patterns and attitudes toward virtual volunteering. Most respondents have used a computer, a smartphone, and the Internet before at home (90.3%), but 22.8% of respondents feel uncomfortable or very uncomfortable when using the Internet. Video conferencing software such as Zoom or Skype was not used before by 14.0% of the respondents, the top reasons being because they prefer other forms of communication (48.4%) or find it too difficult to keep up with technology (19.4%). If tutoring becomes virtual-only, 60.6% of the participants responded they were somewhat likely or very likely to participate, with significant variation by school districts (X2 = 21.92, p < .05, Cramer’s V = 0.33) ranging from 42.6% to 96.0% (Bonferroni post hoc p < .05). Tutors from school districts that were less likely to tutor virtually had lower levels of education and higher levels of discomfort when using the Internet. The respondents also voiced that while virtual tutoring may eliminate barriers to in-person tutoring, such as commuting to schools and inclement weather, they were concerned about establishing a personal connection with their students online. These findings suggest that tutors anticipate both benefits and challenges with virtual volunteering and efforts to engage older adults virtually should factor in prior use of technology and variations by geography.

